# Dietary *Lasia spinosa* Thw. improves reproductive performance of aged roosters

**DOI:** 10.3389/fnut.2022.994783

**Published:** 2022-08-29

**Authors:** Yongxing Hong, Lang Zhang, Xingting Liu, Sile Wu, Jian Wen, Haodong Sun, Kui Tian, Xiaoxuan Jia, Yuying Liao, Wanwipa Suthikrai, Theerawat Tharasanit, Yangqing Lu

**Affiliations:** ^1^State Key Laboratory for Conservation and Utilization of Subtropical Agro-bioresources, College of Animal Science and Technology, Guangxi University, Nanning, China; ^2^Guangxi Veterinary Research Institute, Nanning, China; ^3^Research and Development Center for Livestock Production Technology, Faculty of Veterinary Science, Chulalongkorn University, Bangkok, Thailand; ^4^Faculty of Veterinary Science, Department of Obstetrics, Gynecology and Reproduction, Chulalongkorn University, Bangkok, Thailand

**Keywords:** *Lasia spinosa* Thw., reproductive performance, blood metabolites, RNA-seq, aged rooster

## Abstract

The application of artificial insemination is particularly, owing to which breeder animals are considered an important resource in breeding farms. However, the reproductive performance of roosters typically declines with age, and the economic loss experienced by breeders is attributable to this shortened reproductive lifespan. *Lasia spinosa* Thw. (LST) reportedly improved reproductive capacity in male rodents. The objective of this study was to investigate the effects of LST on the reproductive performance of aged roosters. Male Guangxi Partridge chicken (mean weight, 3032.41 ± 34.48 g; age, 500 days; *n* = 72) randomly received the following three dietary treatments: LST0 group (a basal diet), LST2 group (a basal diet with 2% LST powder), and LST4 group (a basal diet with 4% LST powder). Computer-aided sperm analysis revealed that dietary LST supplementation significantly improved semen volume, sperm motility, and concentration. Furthermore, the most potent effects were observed in the treatment group with the administration of 2% LST, which significantly improved the weight of the testes. Hematoxylin-eosin staining revealed the increase in diameter of the seminiferous tubule and height of the seminiferous tubule epithelium possibly caused as a result of LST treatment. A significant increase in fructose and glucose concentrations were observed in the testis and seminal plasma; in addition, a significant increase was observed in the α-glycosidase levels in the testis and spermatozoa. However, the monoaldehyde levels in the spermatozoa appeared to decline significantly. Additionally, the fertility rate increased significantly following 2% LST supplementation. RNA-seq analysis revealed that 34 and 16 unigenes were upregulated and downregulated, respectively, in testicular tissues from roosters that received dietary supplementation of 2% LST. The assigned functions of the unigenes revealed that LST primarily influenced the mechanisms underlying catalytic activity and cellular processes. Kyoto Encyclopedia of Genes and Genomes enrichment analysis suggested that spermatogenesis-related pathways were significantly enriched, including ABC transporters, ribosome biogenesis in eukaryotes, and VEGF, cAMP, and ErbB signaling pathways.

## Introduction

The male-to-female ratio is considerably low in the poultry industry, owing to which breeder roosters play a vital role in flock fertility. This is particularly important since artificial insemination (AI) is utilized throughout the poultry industry. Ejaculate volume, sperm concentration, and percentage of live spermatozoa are considered core indicators of reproductive capacity in roosters (1). However, the reproductive performance of breeder animals is negatively influenced by aging, with these animals typically showing a decline in performance at ~45 weeks of age (2). This decline is accompanied by a remarkable reduction in semen volume, sperm concentration, and motility. Livestock farms experience a considerable economic loss as the decline in fertility leads to a decline in the production of hatching eggs. Therefore, the development of effective strategies to improve the potential fertility of aged breeder roosters is of paramount importance.

The development of a plant source feed or additive can effectively improve reproductive performance in animals. *Lasia spinosa* Thw. (LST) is a perennial herb that grows extensively in moist areas in tropical and subtropical regions (3). Studies have reported that LST contains flavonoids, phenolic compounds, and carotenoids that may exert their antioxidant, growth-promoting, and antihyperlipidemic properties. Furthermore, LST is used as a vitamin and mineral supplement in the Bodo communities (4). In our previous study, dietary supplementation with LST appeared to considerably improve the growth performance in broilers through the amelioration of gut structure and microbiota and by exerting its antioxidant effects on the serum, liver, and breast muscles (5). A study previously demonstrated that LST could be used as a natural source of phytoestrogens and phytoandrogens (6). In their study, Suthikrai et al. (7) reported that the LST rhizome contained testosterone (T) (8). Additionally, a significant increase in sperm count and testis weight was observed in male rats following oral gavage of LST extracts (8). The abovementioned findings highlighted the potential effects of LST in improving the reproductive performance in male animals.

In the present study, all experiments were designed to explore the impact of LST on the reproductive performance of aged roosters. RNA-seq was performed to analyze the gene expression in testicular tissues to elucidate the role of LST in spermatogenesis. The outcomes of the current study provide evidence to facilitate the development of a sustainable strategy in order to improve the potential fertility of aged roosters and the economic throughput of the poultry industry.

## Materials and methods

### Animal ethics

All procedures used in this study were approved by the Institutional Animal Care and Use Committee of Guangxi University (GXU2018-003).

### Preparation of the plant

LST rhizomes were purchased from a local herbal medicine market (Chongzuo, Guangxi, China) and were washed and dried in the open air without sunlight. The dried rhizomes were then ground to a powder and stored at room temperature (~27°C) until further use.

### Experimental bird and diet

We individually weighed the breeder roosters (Guangxi Partridge chicken; *n* = 72; age, 500 days; initial body weight, 3,032.41 ± °C 34.48 g; Supplementary Figure 1) and randomly assigned them into three experimental groups. The experimental groups were as follows: (1) the LST0 group received a basal diet; (2) the LST2 group received a basal diet with 2% LST powder; (3) the LST4 group received a basal diet with 4% LST powder. The roosters were housed in individual wire cages (70 × 60 × 75 cm) under the same management conditions (27°C and a 15L:9D photoschedule). The roosters were fed a commercial diet supplemented with different amounts of LST powder for 44 days (adaptation period, 7 days; experimentation period, 37 days) with *ad libitum* access to feed and water. The dietary composition and nutrient levels (Table 1) were formulated to meet the recommended nutrient requirements stipulated by the Nutrient requirements of poultry (1994). The ingredients and nutrient contents of LST are shown in Supplementary Table 1 (5).

**Table 1:** Ingredient composition and calculation of ingredients in the basal diet.

**Item**	
**Ingredients (%)**	**Content (g·kg^−1^)**
Corn	680
43 Soybean meal	111
Wheat bran	150
Fish meal	20
CaCO_3_	6
Ca(OH)_2_	10
Poultry mineral substance^a^	1.2
Choline chloride	1
Lysine	2
Methionine	1.4
NaCl	2.3
NaOH	1.5
Total	100
**Calculated analysis** ^**b**^	
Metabolizable energy (MJ/kg)	12.25
Crude protein (%)	14
Methionine (%)	0.24
Calcium (%)	2
Available phosphorus (%)	0.5
Sodium chloride (%)	0.8
Crude fiber (%)	6.5
Crude ash (%)	10

^a^Provides the following components per kg of Diet: Cu, 8 mg; Zn, 75 mg; Fe, 80 mg; Mn, 100 mg; Se, 0.15 mg; I, 0.35 mg.

^b^Nutrient levels were calculated values.

### Semen collection and evaluation

After the adaptation period, semen samples from all roosters were individually collected every 2 days from day 1 of the experimentation period *via* an abdominal massage (9). Semen volume was measured using a 1.5 mL Eppendorf tube. On days 1 and 37, sperm concentration, viability, progressive motility, and motility parameters were measured using an HTM-IVOS II (Hamilton Thorne Biosciences, Beverly, MA, USA). The sperm was considered progressively motile if the progressive average path velocity (VAP) was >40 μm/s and the progressive straightness index (STR) was >80%. Semen quality analyses were performed with eight views and a minimum of 500 sperm tracks were recorded from each sample chamber.

### Fertility rate

A total of 300 healthy 45-week-old hens (Guangxi Partridge) with close laying rates were randomly allocated into three groups for AI. According to the standard process of this commercial farm, for 3 consecutive days, we collected semen from all roosters included in the present study. The semen samples were then mixed evenly and a pipette was used to extract 20 μL of semen for AI. We then began collecting the fertilized eggs every day. After 6 days, another AI was performed. Approximately 650 eggs were collected per group and tested for fertility rate by professional personnel. The measurement of fertility rate was performed on days 1 and 37.

### Blood and testes collection

On day 37, blood samples were collected from the wing vein (six birds per treatment), and centrifugation (3,000 × g) was performed at 4°C for 10 min to separate the serum. The serum was stored at −80°C until further utilization. The testes were removed *via* open surgical castration after slaughter and washed in PBS (pH 7.4, 37°C). Testes weights were measured and the testicular index was determined using the formula reported by Franca et al. (10). The left testes were then immediately stored at −80°C to analyze gene expression and biochemical metabolites, whereas the right testes were fixed in Bouin's solution (10%; pH 7.4) for histological analyses.

### Biochemical analysis of serum and semen

Biochemical components of serum were measured with commercial kits (URIT Medical Electronic Co., Ltd., Guilin, China) and performed using a URIT-8021AVet Auto-Blood Biochemical Analyzer (URIT Medical Electronic Co., Ltd., Guilin, China) according to the manufacturer's instructions. Serum ion levels were measured through an atomic absorption spectrophotometer (URIT Medical Electronic Co., Ltd., Guilin, China). Testosterone levels in the serum were measured using an ARCHITECT i2000sr automatic immunoluminescence analyzer (Abbott Laboratories, IL, USA) according to the manufacturer's instructions.

Fructose in the seminal plasma and testis was measured using a commercial kit (Nanjing Jiancheng Bioengineering, Nanjing, China). α-glycosidase in the testis and spermatozoa was measured using a commercial ELISA kit according to the manufacturer's instructions (Shanghai Enzyme-linked Biotechnology, Shanghai, China). Glucose in the seminal plasma and testis was measured using a URIT-8021AVet Auto-Blood Biochemical Analyzer (URIT Medical Electronic, Guilin, China) according to the manufacturer's instructions. We analyzed the spermatozoa by removing seminal plasma, and then washed the spermatozoa pellet three times with 0.9% sodium chloride solution and suspended it in PBS to achieve a final concentration of 1 × 10^9^ spermatozoa/mL. The spermatozoa were analyzed for malondialdehyde (MDA) using a commercial kit (Nanjing Jiancheng Bioengineering, Nanjing, China).

### Testicular histology

Testicular tissues were collected and submerged in buffered formalin (10% and pH 7.4) for 24 h before dehydrating and fixing with paraffin in a 2T-12M tissue processor (XiaoganYaguang Medical Electronic, Xiaogan, China). The fixed tissues were embedded in paraffin blocks using an embedding system (Leica, Germany). The sections were then sliced into a diameter of 5 μm using a rotary microtome (Leica, Germany) and further processed for hematoxylin-eosin (HE) staining. The seminiferous tubule diameter (STD) and seminiferous tubule epithelial height (SEH) were measured using an EVOS™ M5000 Imaging System (ThermoFisher Scientific, MA, USA). STD was defined as the shortest distance between the outer edge of the tubule and the SEH between the germ cells.

### RNA-Seq analysis

Total RNA was isolated from six testes (three LST0 and three LST2), and the RNA integrity was determined using a 2100 Bioanalyzer Instrument (Agilent Technologies, CA, USA). Sequencing libraries were generated using the VAHTS Universal V6 RNA-seq Library Kit for MGI (Vazyme, Nanjing, China) according to the manufacturer's recommendations, and index sequences were added to attribute sequences to each sample. RNA sequencing was performed on an MGI-SEQ 2000 platform to create 150 bp paired-end reads. Differentially expression genes (DEGs) between the LST0 and LST2 groups were evaluated *via* DESeq2 (11). The false discovery rate (FDR) was used to identify the threshold of the *P*-value in multiple tests to compute the significance of the differences. Herein, only genes with logarithmic values of expression fold-changes |log2(FoldChange)| ≥1 and FDR significance score (padj) <0.05 were used for subsequent analyses.

### Functional annotation

We compared DEGs against the NCBI nr database and the Swiss-Prot database for their functional annotations. Gene Ontology (GO) annotation was performed based on the correspondence between genes in NCBI and their GO annotations. The database of this correspondence was obtained from the following URL: https://ftp.ncbi.nlm.nih.gov/gene/DATA/gene2go.gz. Kyoto Encyclopedia of Genes and Genomes (KEGG) pathway annotation was performed using BLASTx. GO and KEGG enrichment was performed using the hypergeometric test as implemented in the R phyper function.

### Quantitative reverse transcriptase-polymerase chain reaction (qRT-PCR)

Total RNA of testes was extracted using a Total RNA Kit I (OMEGA Bio-Tek, GA, USA) and reverse transcribed to cDNA using a TransScript II One-Step gDNA Removal and cDNA Synthesis SuperMix kit (TransGen Biotech, Beijing, China). The primer sequences of the selected genes are shown in Supplementary Table 2. Quantitative PCR assays were performed using the PerfectStart Green qPCR SuperMix (TransGen Biotech, Beijing, China). Each experiment was performed in triplicates.

### Statistical analysis

The IBM SPSS Statistics V23.0.0 software (SPSS Inc., Chicago, IL, USA) was used to perform all statistical analyses. We analyzed data from days 1–37 in the same treatment group in addition to gene expression with a *t*-test. Statistically significant differences among the three treatments were tested using one-way analysis of variance (ANOVA) with a Fisher's Least Significant Difference (LSD) Test. The results were expressed as mean and standard error of the mean (SEM). Statistical significance was indicated by *P* < 0.05.

## Results

### Effects of dietary LST powder supplementation on the semen volume and sperm quality of aged roosters

**Figure 1:**
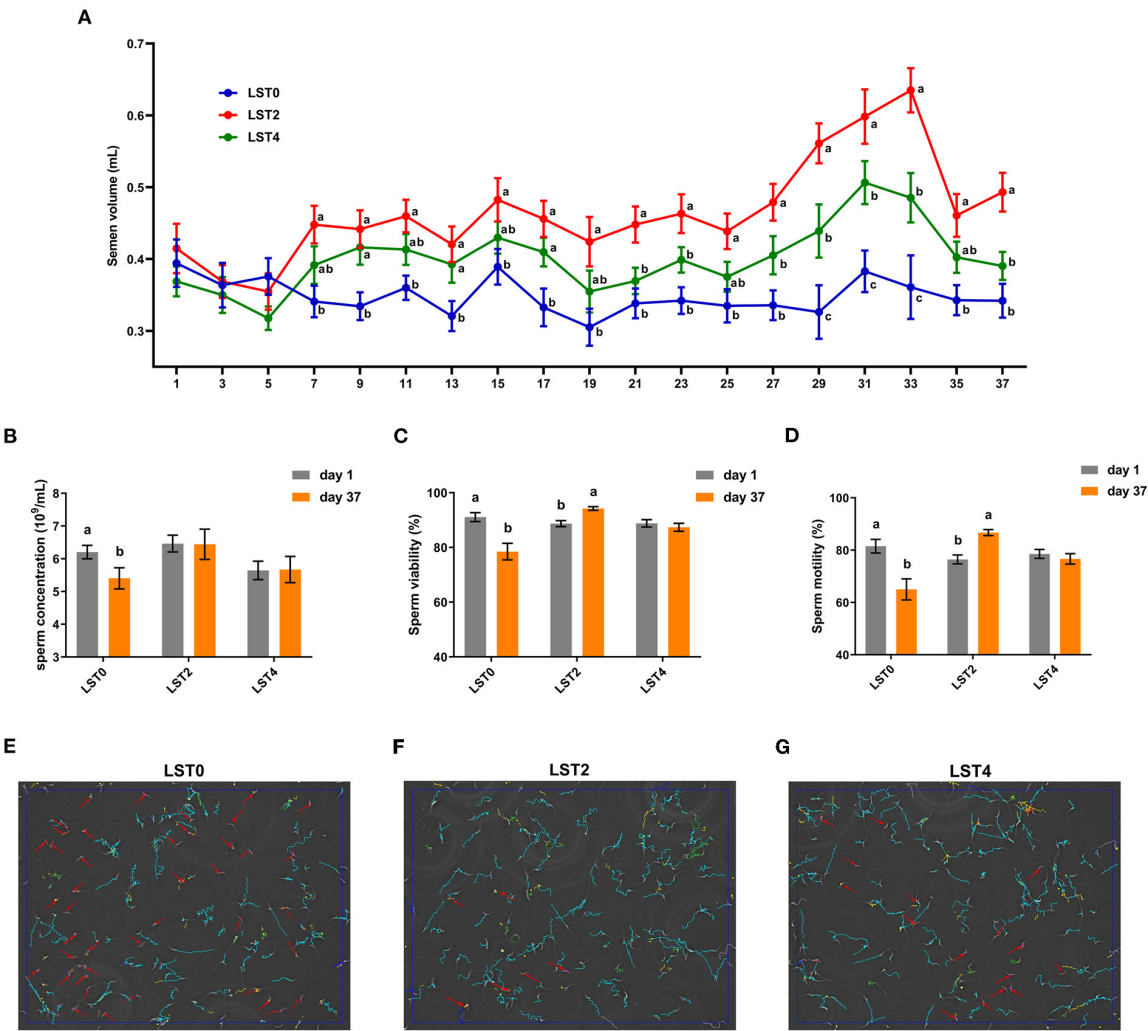
Effects of dietary *Lasia spinosa* Thw. (LST) powder supplementation on semen volume and quality of aged roosters. **(A)** Dynamics of semen volume after LST treatment (*n* = 24). Mean values within the same abscissa with different letters showed significant differences (*P* < 0.05). **(B)** Sperm concentration after LST treatment (*n* = 10). **(C)** Sperm viability after LST treatment (*n* = 10). **(D)** Sperm motility after LST treatment (*n* = 10). **(E–G)** Representative sperm analysis figures of in the LST0, LST2, and LST4 groups, respectively. The red arrow represents a sperm being slowly motile or dead. The blue route represents a sperm being progressively motile. The green route represents a sperm being viable.

The semen volume and sperm quality were measured at the start of the experiment and showed no significant differences among the roosters between the treatment and control groups (Figure 1A). From the 7th day after treatment, the volume of semen from roosters in the LST2 and LST4 groups was significantly higher than that in the LST0 group. Semen volume in the LST2 and LST4 groups was highest on days 33 and 31 after treatment (0.63 and 0.51 mL per rooster), respectively, and these values were significantly (*P* < 0.001) higher than those observed in the LST0 group. In addition, sperm concentration in the LST0 group significantly decreased on day 37 than that recorded on day 1 (*P* = 0.036, Figure 1B), whereas those in the LST2 and LST4 groups remained relatively stable (*P* > 0.05). Similarly, sperm viability and motility (Figures 1C,D) in the LST0 group decreased significantly (*P* < 0.01) at the end of the experiment, whereas those in the LST2 group increased significantly (*P* < 0.01), and those in the LST4 group showed no significant difference when compared with the values measured on day 1 (*P* > 0.05). The representative sperm analysis pictures and videos of each group at the end of this experiment are presented in Figures 1E–G and Supplementary Videos 1–3, respectively.

To further explore the effects of dietary LST supplementation on sperm motility in aged roosters, we comprehensively assessed the motility parameters. As shown in Table 2, the VAP, VCL, VSL, ALH, LIN, and WOB were significantly decreased in the LST0 group when compared with the values measured on day 1 (*P* ≤ 0.01); however, sperm mobility remained relatively steady in the LST2 and LST4 (*P* > 0.05) except for WOB (*P* = 0.043) in the LST4 group.

**Table 2:** Effects of dietary LST powder supplementation on sperm motility of aged roosters.

**Item^a^**		**Treatment** ^**b**^		
		**LST0**	**LST2**	**LST4**
VAP, μm/s	Day 1	81.67 ± 4.82	77.75 ± 3.06	76.86 ± 3.01
	Day 37	61.17 ± 2.99	85.59 ± 3.80	71.42 ± 3.31
	*P*-value	0.002	0.126	0.24
VCL, μm/s	Day 1	121.94 ± 6.01	117.87 ± 4.02	116.79 ± 3.60
	Day 37	98.02 ± 3.65	128.52 ± 3.84	113.96 ± 3.82
	*P*-value	0.003	0.072	0.597
VSL, μm/s	Day 1	66.59 ± 4.48	61.67 ± 3.15	62.83 ± 2.74
	Day 37	48.53 ± 2.80	70.17 ± 4.23	57.17 ± 3.20
	*P*-value	0.003	0.124	0.195
ALH, %	Day 1	7.47 ± 0.28	7.27 ± 0.15	6.95 ± 0.19
	Day 37	6.21 ± 0.17	7.53 ± 0.21	6.87 ± 0.16
	*P*-value	0.001	0.324	0.756
BCF, Hz	Day 1	25.77 ± 0.41	26.62 ± 0.37	26.54 ± 0.31
	Day 37	27.26 ± 0.62	25.80 ± 0.48	27.09 ± 0.32
	*P*-value	0.061	0.194	0.239
LIN, %	Day 1	52.99 ± 1.25	50.54 ± 1.17	52.08 ± 0.89
	Day 37	47.54 ± 1.43	53.74 ± 1.98	49.22 ± 1.22
	*P*-value	0.01	0.18	0.075
STR, %	Day 1	78.13 ± 0.92	76.37 ± 0.78	78.28 ± 0.61
	Day 37	75.91 ± 0.81	79.07 ± 1.46	77.11 ± 0.94
	*P*-value	0.085	0.12	0.306
WOB, %	Day 1	65.59 ± 0.91	63.68 ± 0.81	64.29 ± 0.70
	Day 37	60.51 ± 1.32	65.81 ± 1.33	61.65 ± 0.99
	*P*-value	0.005	0.188	0.043

^a^VAP, Average Path Velocity; VCL, Average Curvilinear Velocity; VSL, Average Straight-Line Velocity; ALH, Amplitude of Lateral Displacement of Sperm Head; BCF, Beat/Cross Frequency; LIN, Linearity Index [(VSL/VCL) × 100]; STR, Straightness Index [(VSL/VAP) × 100]; WOB, Wobble Index [(VAP/VCL) × 100].

^b^LST, *Lasia spinosa* Thw.; LST0, control diet; LST2, 2% supplementation level of *Lasia spinosa* Thw. powder; LST4, 4% supplementation level of *Lasia spinosa* Thw. powder. Values are means ± SEM (n = 10).

### Effects of dietary LST supplementation on testicular weight and morphology of aged roosters

**Figure 2:**
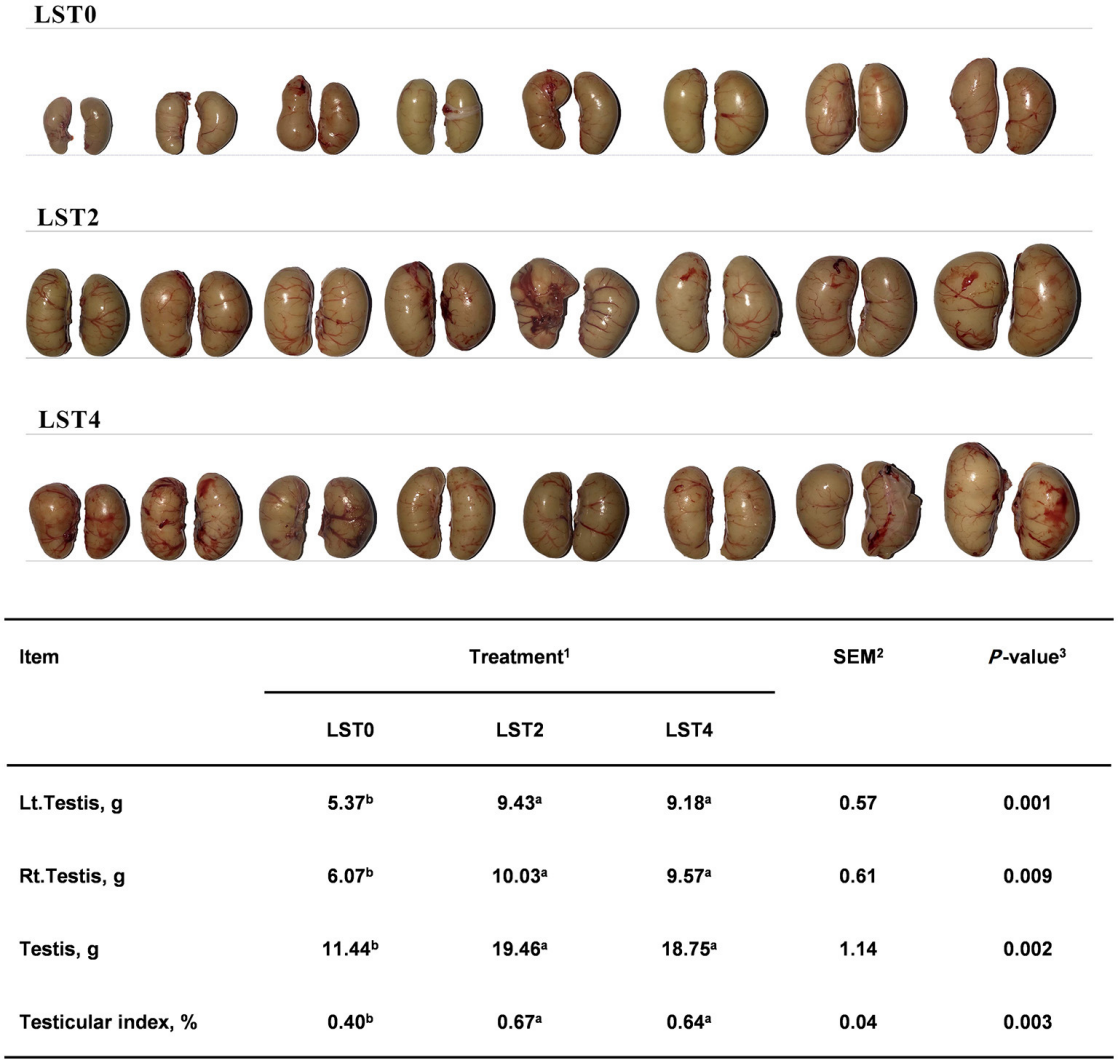
Effects of dietary LST powder supplementation on the testicular weight of aged roosters. ^1^LST, *Lasia spinosa* Thw.; LST0, control diet; LST2, dietary supplementation of 2% LST powder; LST4, dietary supplementation of 4% LST powder. ^2^SEM, standard error of the mean. ^3^Mean values within a row with different letters differ at *P* < 0.05 (*n* = 8).

To determine the effects of LST on the testis, the weight and morphology of the testis were examined in aged roosters at the end of the treatment period. As shown in Figure 2, the testicular weight and testicular index after LST supplementation were significantly higher (*P* < 0.01) than those in the LST0 group.

**Figure 3:**
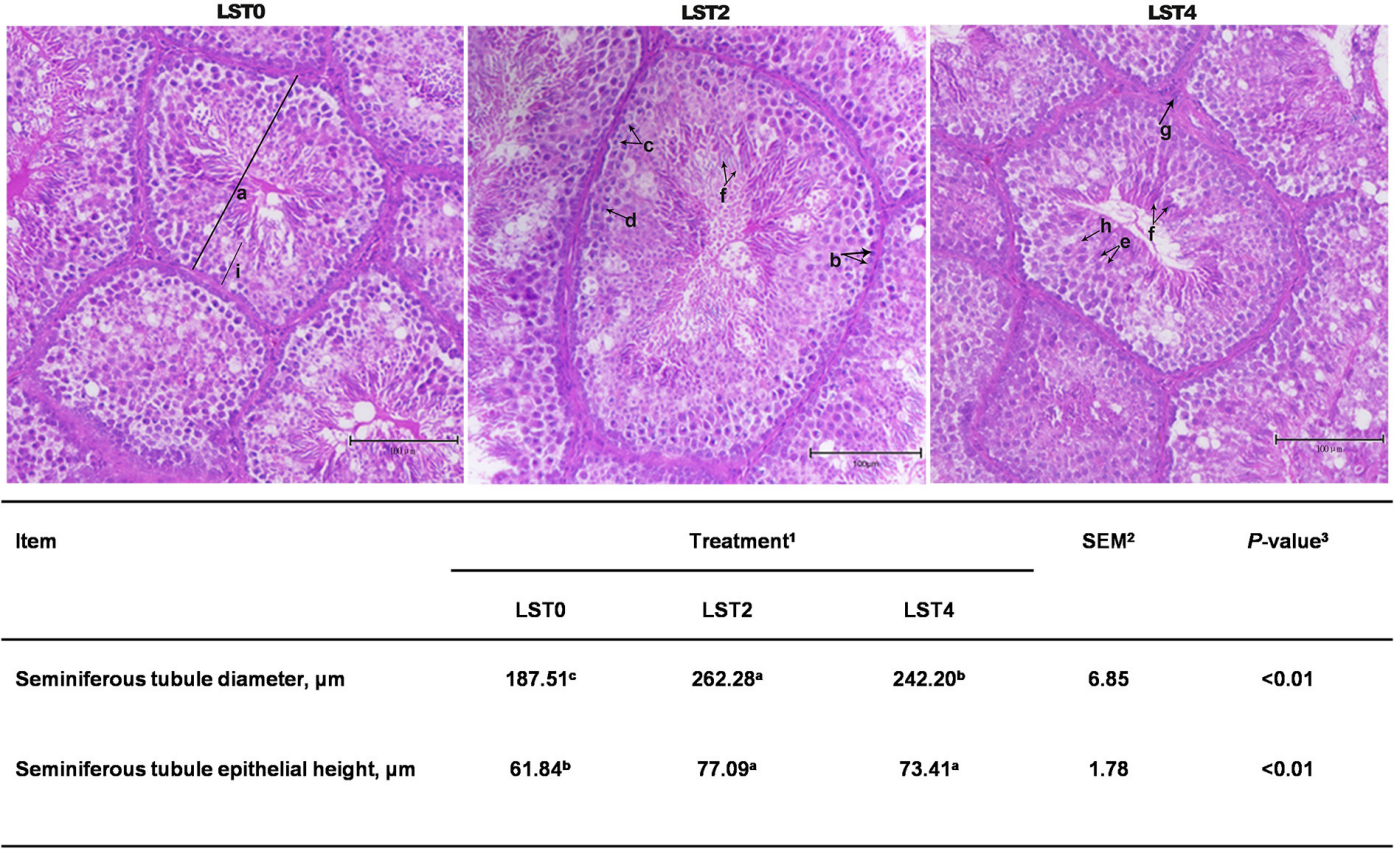
Testicular histology of aging roosters. (a) Seminiferous tubule diameter. (b) Spermatogonia. (c) Primary spermatocyte. (d) Secondary spermatocyte. (e) Spermatid. (f) Spermatozoon. (g) Leydig cell. (h) Sertoli cell. (i) Seminiferous tubule epithelial height. Scale bars, 100 μm. ^1^LST, *Lasia spinosa* Thw.; LST0, control diet; LST2, dietary supplementation of 2% LST powder; LST4, dietary supplementation of 4% LST powder. ^2^SEM, standard error of the mean. ^3^Mean values within a row with different letters differ at *P* < 0.05.

HE staining was performed to examine the morphology of the testis. Figure 3 shows that STD and SEH in the LST2 and LST4 groups were significantly higher (*P* < 0.01) than those in the LST0 group. In the LST2 group, in particular, the testes exhibited significantly higher (*P* < 0.01) STD values than those in the LST0 and LST4 groups.

### Effects of dietary LST supplementation on the metabolites and mineral profile in the serum

The effects of dietary LST treatment on the physical status of aged roosters were measured by examining the blood metabolites and mineral profiles. Our results revealed that there were no significant differences in terms of the blood metabolites except for triglyceride, cholesterol, and LDL_C levels (Table 3). The levels of triglyceride and LDL_C were significantly (*P* < 0.05) reduced in the LST treatment groups. Furthermore, the LST2 group showed significantly reduced cholesterol levels (*P* = 0.018). The Ca and Fe levels were significantly (*P* < 0.05) increased in roosters fed with dietary LST.

**Table 3:** Blood metabolites and mineral profile of aged roosters after dietary supplementation of LST powder.

**Item^1^**	**Treatment** ^**2**^			**SEM**	***P*-value^3^**
	**LST0**	**LST2**	**LST4**	
**Blood metabolites**					
ALT, U/L	2.17	2.17	2.33	0.19	0.927
AST, U/L	228	232.17	228.67	12.6	0.991
Total protein, g/L	54.75	53.97	52.83	0.84	0.675
Albumin, g/L	19.6	20.55	19.58	0.28	0.29
DBIL, μmol/L	4.42	4.67	4.93	0.28	0.771
TBIL, μmol/L	80.88	82.23	84.1	1.08	0.502
Urea, mmol/L	0.83	0.9	0.9	0.02	0.216
Uric Acid, μmol/L	441.17	420.83	440.67	17.6	0.879
Triglyceride, mmol/L	2.1^a^	1.57^b^	1.65^b^	0.09	0.027
Cholesterol, mmol/L	4.05^a^	3.45^b^	3.74^ab^	0.09	0.018
HDL_C, mmol/L	1.16	1.11	1.18	0.04	0.797
LDL_C, mmol/L	1.02^a^	0.84^b^	0.86^b^	0.03	0.01
Glucose, mmol/L	12.11	12.12	12.38	0.11	0.532
RF, IU/mL	3.35	3.4	3.25	0.05	0.552
**Mineral profile**					
Calcium, mmol/L	7.27^c^	8^a^	7.6^b^	0.08	<0.01
Iron, μmol/L	28.23^b^	36.02^a^	33.9^a^	1.19	0.011
Magnesium, mmol/L	1.09	1.1	1.14	0.02	0.573
Phosphorus, mmol/L	1.33	1.28	1.27	0.02	0.554

^1^ALT, Alanine Aminotransferase; AST, Aspartate Aminotransferase; DBIL, Direct Bilirubin; TBIL, Total Bilirubin; HDL-C, High-Density Lipoprotein Cholesterol; LDL-C, low-Density Lipoprotein Cholesterol; RF, Rheumatoid Factors.

^2^LST, *Lasia Spinosa* Thw.; LST0, Control Diet; LST2, 2% Supplementation Level of *Lasia Spinosa* Thw. powder; LST4, 4% Supplementation Level of *Lasia Spinosa* Thw. powder.

^3^SEM, standard error of the mean.

### Effects of dietary LST supplementation on biochemical properties in testis, seminal plasma, sperm, and fertility rate after dietary LST supplementation

**Figure 4:**
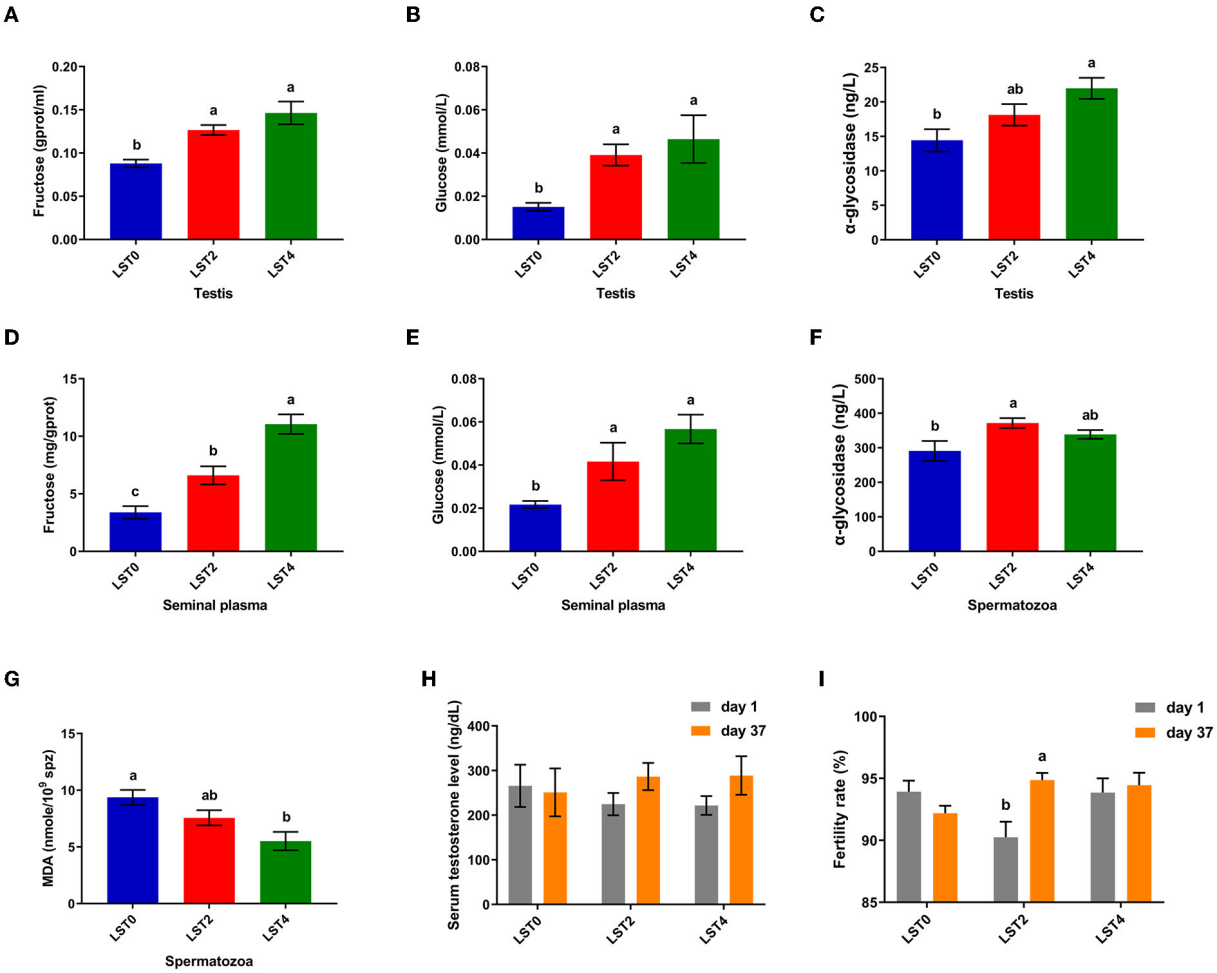
Effects of dietary LST supplementation on biochemical indices of testis and sperm of aged roosters. **(A)** The fructose level in the testis after LST supplementation (*n* = 6). **(B)** The glucose level in the testis after LST supplementation (*n* = 6). **(C)** The α-glycosidase level in the testis after LST supplementation (*n* = 6). **(D)** The fructose level in the seminal plasma after LST supplementation (*n* = 6). **(E)** The glucose level in the seminal plasma after LST supplementation (*n* = 6). **(F)** The α-glycosidase level in the spermatozoa after LST supplementation (*n* = 6). **(G)**The MDA level in the spermatozoa after LST supplementation (*n* = 6). **(H)** The serum testosterone level after LST supplementation (*n* = 10). **(I)** The fertility rate after LST supplementation. Values are means ± SEM. a, b, c: *P* < 0.05.

To elucidate the mechanism through which LST improved sperm motility, we examined the biochemical indices of the testes and sperms. Figure 4 shows that fructose and glucose levels in the testis in the LST treatment groups increased significantly (*P* < 0.05), and the α-glycosidase level of testis in the LST4 group was significantly (*P* = 0.004) higher than that in the LST0 group. Moreover, fructose and glucose levels of seminal plasma (Figures 4D,E) after LST supplementation were (*P* < 0.01) higher than that of the control group, whereas the α-glycosidase level of spermatozoa in the LST2 group increased significantly (*P* = 0.012) (Figure 4F). Regarding lipid peroxidation levels, the MDA level (Figure 4G) of spermatozoa in the LST4 groups decreased significantly (*P* = 0.002) compared with the LST0 group. However, no significant (*P* > 0.05) difference was observed in terms of testosterone levels (Figure 4H) in the treatment and control groups. Furthermore, the fertility rate in the LST2 group improved significantly (Figure 4I), highlighting the effectiveness of the LST intervention.

### Effects of dietary LST supplementation on the transcriptional profile of testicular tissues

**Figure 5:**
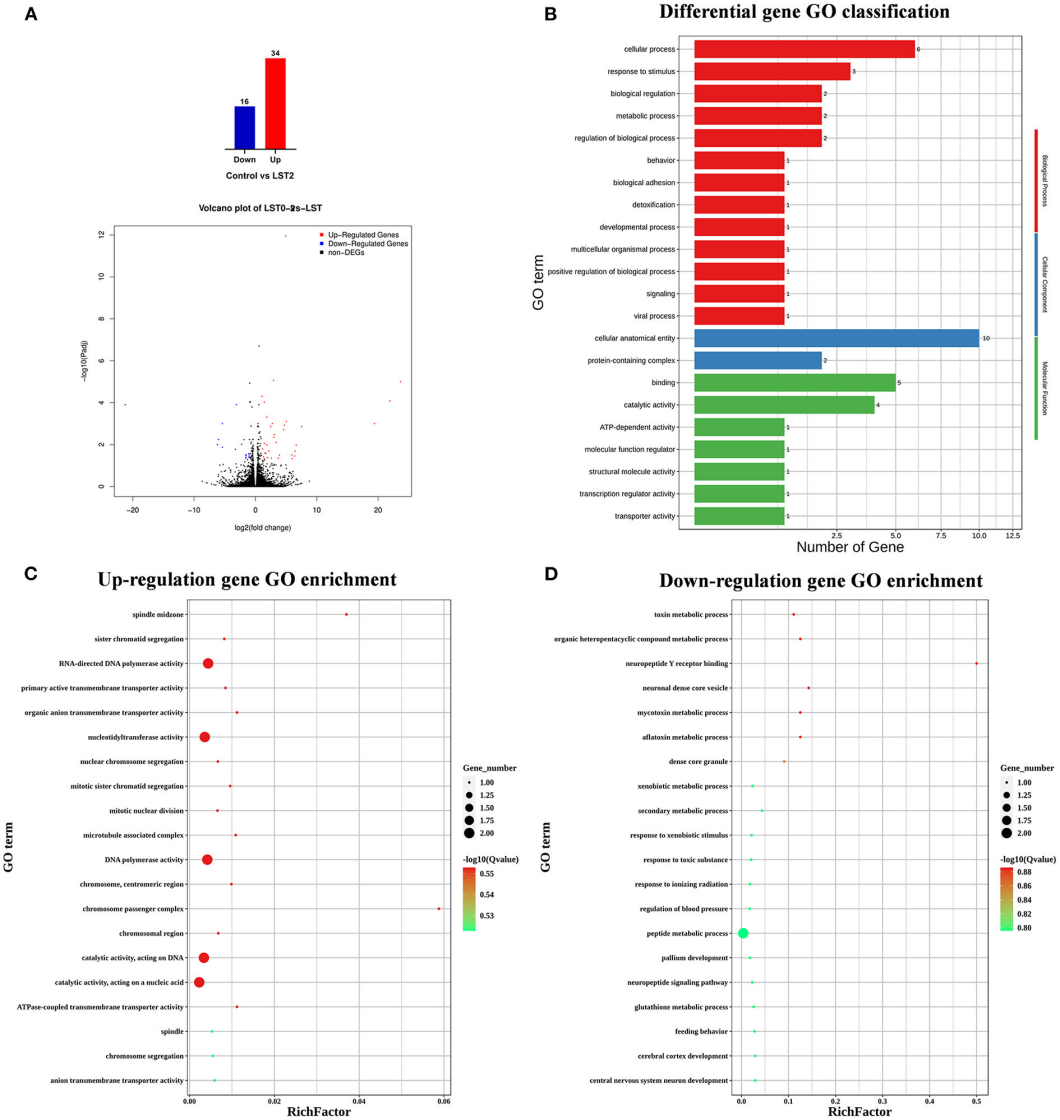
Transcriptome analysis of testis after LST treatment. **(A)** The number of DEGs and volcano plot analysis between control and LST2 treatments. **(B)** GO classification of DEGs between control treatment and LST2 treatment. **(C)** The rich factor according to the upregulation gene GO enrichment analysis for the DEGs after LST treatment. **(D)** The rich factor according to the downregulation gene GO enrichment analysis for the DEGs after LST treatment.

**Figure 6:**
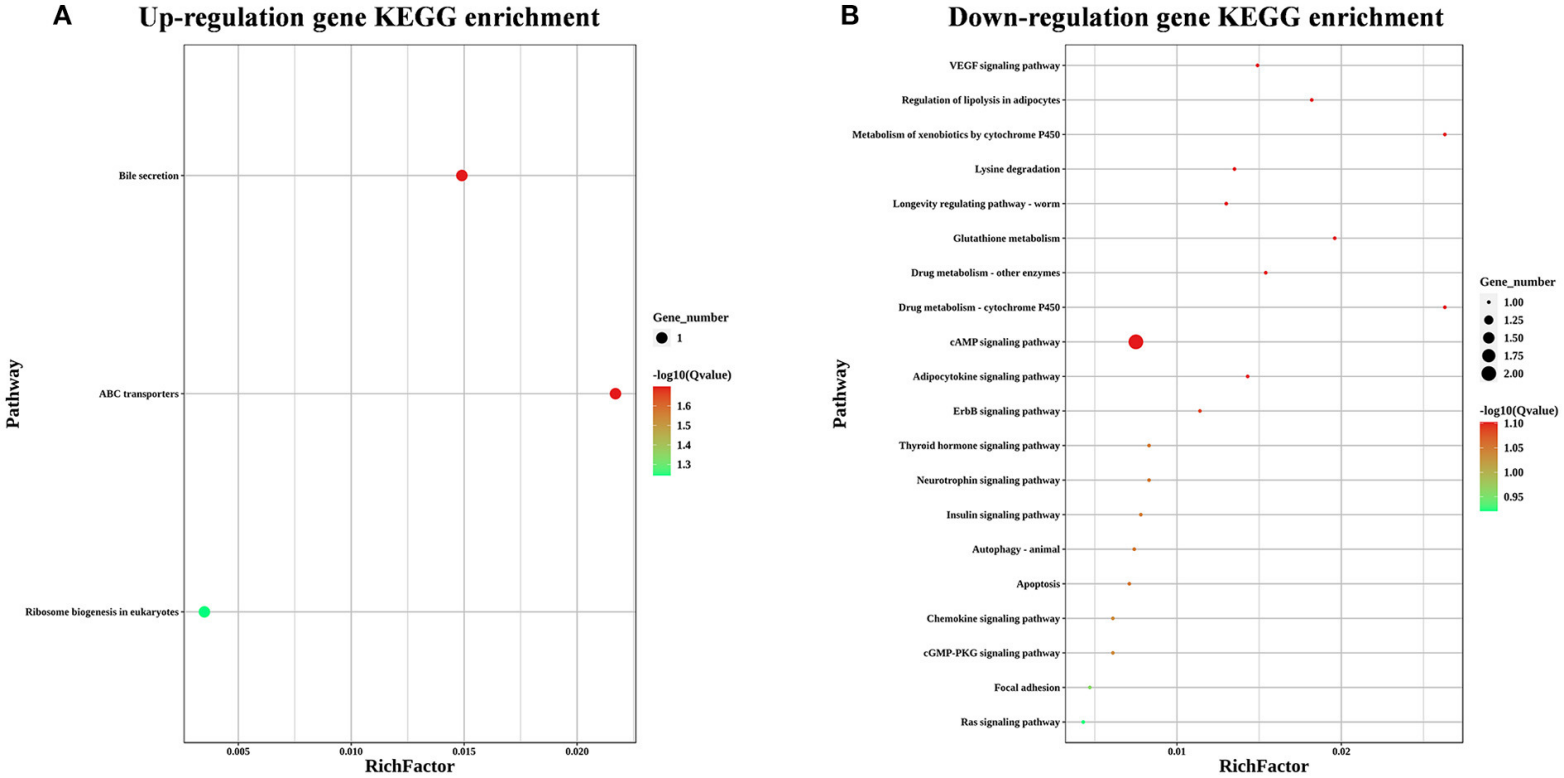
KEGG enrichment analysis for the differentially expressed genes after LST treatment. **(A)** KEGG enrichment analysis for the upregulated genes after LST treatment. **(B)** KEGG enrichment analysis for the downregulated genes after LST treatment.

To explore the effects of LST on gene expression in testis, RNA-seq was performed for testes from the LST0 and LST2 groups at the end of the experiment. Figure 5A shows that, through transcriptome analysis, 34 were upregulated and 16 were downregulated in the LST2 treatment group (Figure 5A). GO analysis revealed that most DEGs were enriched in terms of the biological processes and LST affected the function of catalytic activity and cellular process (Figure 5B). The GO term functional enrichment database shown in Figure 5C indicated that the upregulated DEGs were significantly involved in intracellular anatomical structure, cell cycle, mitosis, catalytic activity, transmembrane transporter activity, and nucleotidyl transferase activity. As shown in Figure 5D, the downregulated DEGs were primarily involved in the metabolic processes. The KEGG pathway functional enrichment database shown in Figure 6A indicated that the upregulated DEGs were involved in bile secretion, ATP-binding cassette (ABC) transporter, and ribosome biogenesis in eukaryotes pathways. Additionally, the KEGG enrichment analysis showed that several genes were downregulated in testis after LST supplementation; these genes included VEGF, cAMP, ErbB, insulin, thyroid hormone, autophagy, and apoptosis pathways (Figure 6B). These results provided general information related to the functional pathway for the effects of LST supplementation on testes.

### Validation of differential expressed genes by qRT-PCR

**Figure 7:**
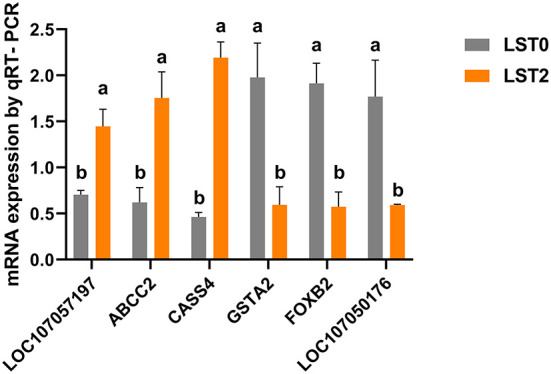
Validation of RNA-seq results with quantitative reverse transcriptase-polymerase chain reaction (qRT-PCR) analysis. The mRNA levels of selected genes were analyzed using qRT-PCR and normalized to β-actin. *ABCC2*, ATP binding cassette subfamily C member 2; *CASS4*, Cas scaffold protein family member 4; *GSTA2*, glutathione S-transferase alpha 2; *FOXB2*, forkhead box B2. All values are expressed as means ± SEM (*n* = 3). a, b: *P* < 0.05.

We verified the reliability of RNA-seq results by validating the expression of 3 upregulated genes (*LOC107057197, ABCC2*, and *CASS4*) and 3 downregulated genes (*GSTA2, FOXB2*, and *LOC107050176*) *via* qRT-PCR. The expression levels of both the upregulated and downregulated groups of genes were consistent with the tendency of the RNA-seq results (Figure 7).

## Discussion

Aging is closely associated with a decline in reproductive performance and fertility, and older males in most species show dysfunctions in the reproductive axis, steroidogenesis, and spermatogenesis as a result of morphological, compositional, and functional alterations in the testes (12). Therefore, prevention or delaying age-associated reproductive disorders would be necessary for the maintenance of fertility in agriculturally important breeder animals.

In the present study, we observed that the sperm concentration decreased significantly in the roosters fed with a commercial diet (control) at day 37; however, those fed with a diet supplemented with LST2 and LST4 did not change when compared with the levels recorded on day 1. These results agreed with the outcomes reported in a previous study considering that LST administration for 7 days increased the sperm count in rats (8). Additionally, there was a significant improvement in the semen volume of roosters in both LST2 and LST4 groups when compared with those in the control group. This improvement in semen volume and sperm concentration highlighted the potent spermatogenesis-promoting capacity of LST. Kaewamatawong et al. proposed that the phytohormone in the LST rhizome was primarily responsible for the observed improvements in reproductive performance (8). Owing to the fact that we observed no significant changes in testosterone levels in the serum of all the groups, and the fact that the correct composition of LST powder is a relatively controversial subject, further investigation is warranted to identify the pertinent chemicals in LST and elucidate their roles in reproductive physiology.

Testes undergo morphological degeneration, which subsequently arrests spermatogenesis in aged animals (13). To elaborate, testicular aging was involved in the thinning of the SEH and a decrease in the STD (14). In the present study, degeneration of the spermatogenic tubules was attenuated through the dietary supplementation of LST, as indicated by the increased STD and SEH levels when compared with those from the control group. The higher levels of SEH could be attributable to the higher number of Sertoli and spermatogonia cells (15). In addition, we observed that the testicular weight and index increased significantly in the LST group, which was similar to a finding reported by Kaewamatawong et al. (8). The increase in testicular weight and index may have been attributable to the increased SEH, the presence of a greater number of Sertoli and spermatogonia cells, which suggested the presence of a higher potential for spermatogenesis in these roosters. These findings support our observation that the ejaculation volume and sperm density of roosters in the LST supplement groups were higher than those of the control group. The process involved in the development of germ cells requires adequate levels of energy substrates; however, if this energy demand is not satisfied, the germ cells will eventually degenerate and enter the apoptotic pathway (16). Developing germ cells preferentially use lactate as a substrate for energy production and Sertoli cells are capable of producing lactate by metabolizing various substrates, glucose in particular (17). In the present study, when compared with the control group, there was a significant increase in glucose and α-glycosidase levels in the testis after LST administration. Therefore, the spermatogenesis capacity of LST treatment groups may be enhanced by ensuring improvements in energy metabolism.

Sperms primarily rely on glycolysis for energy metabolism, with fructose and glucose as the main substrates for energy production (18, 19). Higher fructose levels in seminal plasma typically result in a relatively higher ratio of straight-moving sperm (20). In the present study, the fructose and glucose levels in the seminal plasma and testes in the roosters increased significantly in the LST supplement group. Furthermore, the VAP, VCL, VSL, ALH, LIN, and WOB of sperms from roosters in the control group decreased significantly, whereas the sperm motility in the supplementation groups remained relatively steady throughout the study period. Kinetic parameters (especially VSL and VCL) play a critical role in regulating overall sperm quality (21). Both VSL and VCL are important variables in terms of fertility in animals (22–24) and a higher VSL provides a competitive advantage in sperm competition (25). Moreover, α-glycosidase concentrations in testicular tissue and spermatozoa increased significantly in the LST group when compared with control roosters, thereby suggesting the fact that the sperm metabolism was enhanced and the sperm quality had improved (26).

Mitochondrial oxidation is the primary pathway responsible for the production of energy used for flagellar motion (19). Reactive oxygen species (ROS) are produced in the process of mitochondrial oxidation, and excessive production or accumulation of ROS may result in oxidative stress, thereby leading to DNA damage and lipid peroxidation (27). In aging roosters, the weakened antioxidant defense system makes sperms vulnerable to lipid peroxidation (28); furthermore, their cytoplasm is primarily confined to the mid-segment without enough antioxidants to offer adequate protection against oxidative stress (28, 29). Therefore, diet-derived antioxidants may be critical for eliminating cumulative oxidative damage. A previous study reported that MDA, as an important marker of lipid peroxidation, was negatively correlated with sperm motility (30). In the current study, the MDA content in the spermatozoa appeared to decrease in a dose-dependent manner in the dietary LST treated group. Moreover, there was a significant increase in the levels of superoxide dismutase, catalase, and glutathione peroxidase in a dose-dependent manner in LST treatment groups, whereas the ROS and MDA levels appeared to decrease in the serum, liver, and breast muscle (5). These results implied that the LST functions as a source of antioxidants in roosters. Furthermore, these antioxidant effects may arise from polyphenols, ascorbic acid, flavonoids, and phytosterols enriched in LST (5). Therefore, dietary LST may improve sperm quality by promoting energy metabolism and reduction of peroxidation.

Blood metabolites are common indicators of animal health. A previous report revealed that TG values decreased significantly in male rats that received LST supplementation; however, no obvious toxicological effects were observed in the acute and subchronic terms (8). In the present study, the TG and LDL-C levels decreased significantly in roosters from LST groups, which is consistent with our previous study on young broilers (5). Moreover, the mineral levels of Ca and Fe were significantly increased in the treatment groups. This may partially be attributable to the rich trace element in LST (4). We noted no other difference in the blood metabolite levels in roosters in the LST groups, which suggested that dietary LST has no detrimental impacts or toxicity in the aged roosters.

Spermatogenesis is a complicated process and requires precise regulation of related genes in the niche. GO classification analysis of that gene expression profile in the testis of LST-treated roosters reveals that most upregulated DEGs were enriched during the cell cycle, mitosis, and catalytic activity in the testis after LST supplementation. These findings could be explained by the fact that the mitotic proliferation of spermatogonia, the meiotic division of primary spermatocytes, and the development into round spermatids are the major events that occur during spermatogenesis in the testis (31).

Furthermore, by matching DEGs to the KEGG database, we identified 25 pathways, among which the “ABC transporters,” “Ribosome biogenesis in eukaryotes,” “VEGF signaling pathway,” “cAMP signaling pathway,” and “ErbB signaling pathway” were closely associated with reproductive performance. The family of ABC transporters is critical in transporting the nutrients and other molecules into cells (32); in addition, several members are reportedly involved in the processes underlying mitosis and meiosis of germ cells through the transport of nucleotides essential for the development of germ cells (33). *ABCC2* was significantly upregulated in testes of roosters after LST supplementation (34). Ribosome biogenesis can lead to the formation of numerous proteins during the development of the testes and spermatogenesis (35). These results indicated that LST supplementation may promote spermatogenesis by improving nutrition metabolism. Dysregulation of VEGF can suppress spermatogonia proliferation and spermatogenesis, which results in male infertility (36). Don and Stelzer demonstrated that the cAMP-dependent signaling pathway, as a crucial regulatory mechanism, functions at different stages of spermatogenesis (37). ErbB signaling component genes play an important part in regulating spermatogenesis (38). These results suggested that LST administration may enhance spermatogenesis through the regulation of signal transduction.

## Conclusions

In conclusion, our current study shows that the reproductive performance of aged roosters improved significantly after dietary supplementation of 2% LST. The spermatogenesis of aged roosters may be improved *via* regulation of signal transduction and energy metabolism and amelioration of oxidative stress by administration of chemicals from LST. Further investigation is warranted to identify the chemicals in LST and their underlying mechanism related to the promotion of reproduction. Results of the present study provide evidence for the development of new plant-source additives dietary to promote reproductive performance in roosters.

## Data availability statement

The datasets presented in this study can be found in online repositories. The names of the repository/repositories and accession number(s) can be found below: https://www.ncbi.nlm.nih.gov/, PRJNA853084.

## Ethics statement

The animal study was reviewed and approved by the Institutional Animal Care and Use Committee of Guangxi University (GXU2018-003).

## Author contributions

YxH: formal analysis and writing-original draft. XtL: writing - review. SlW, JW, HdS, KT, LZ, YxH, and XxJ: investigation. YyL: project administration. WS and TT: conceptualization. YqL: writing - conceptualization, review and editing, and funding acquisition. All authors read and approved the final manuscript.

## Funding

This study was jointly supported by the National Natural Science Foundation of China (31960157), the National Key R&D Program of China (2021YFD1300100), and Guangxi Key R&D Program (AB21220005).

## Conflict of interest

The authors declare that the research was conducted in the absence of any commercial or financial relationships that could be construed as a potential conflict of interest.

## Publisher's note

All claims expressed in this article are solely those of the authors and do not necessarily represent those of their affiliated organizations, or those of the publisher, the editors and the reviewers. Any product that may be evaluated in this article, or claim that may be made by its manufacturer, is not guaranteed or endorsed by the publisher.

## Supplementary material

Supplementary Figure 1 Effects of dietary LST powder supplementation on the body weight of aged breeder roosters.

Supplementary Table 1 Ingredients and nutrient contents of LST.

Supplementary Table 2 Primers used for Real-time PCR.

Supplementary Video 1 Representative sperm analysis video of the LST0 group at the end of this experiment.

Supplementary Video 2 Representative sperm analysis video of the LST0 group at the end of this experiment.

Supplementary Video 3 Representative sperm analysis video of the LST0 group at the end of this experiment.
