# Supplementary figures and images for: Dietary Lasia spinosa Thw. improves reproductive performance of aged roosters
Source: Front Nutr. 2022 Aug 29;9:994783. doi: 10.3389/fnut.2022.994783 (PMC9466466; doi:10.3389/fnut.2022.994783)

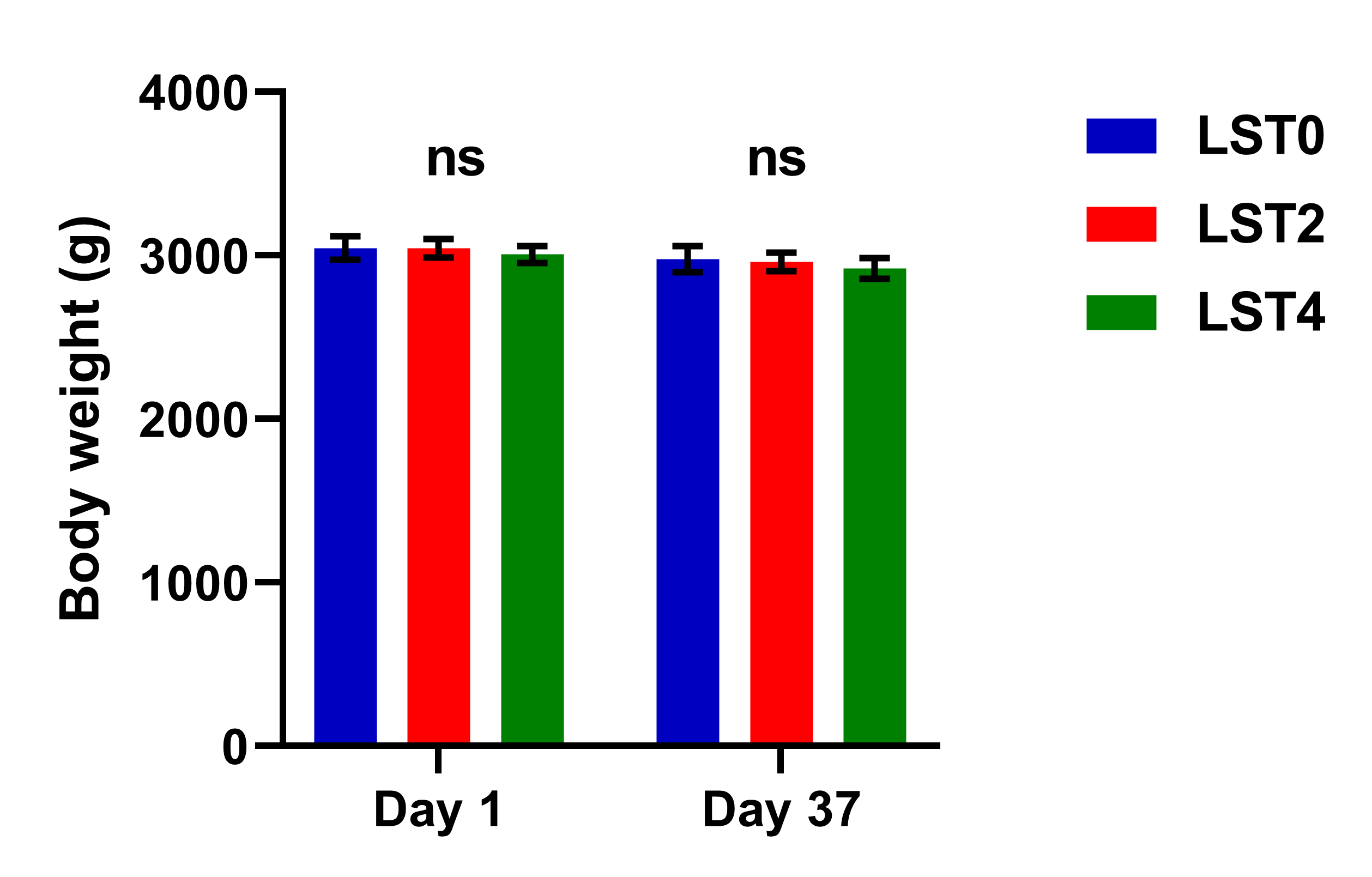

Supplement: Supplementary Figure 1 — Effects of dietary LST powder supplementation on the body weight of aged breeder roosters. [file Image_1.TIF]
