# Supplementary material for: Dietary Lasia spinosa Thw. improves reproductive performance of aged roosters
Source: Front Nutr. 2022 Aug 29;9:994783. doi: 10.3389/fnut.2022.994783 (PMC9466466; doi:10.3389/fnut.2022.994783)
Supplement: Supplementary Table 1 — Ingredients and nutrient contents of LST. [file Table_1.DOCX]

**Table S1**

Ingredients and nutrient contents of LST

| **Components (%)** | **As Sampled** | **Dry Matter Basis** |
| --- | --- | --- |
| [Moisture](javascript:;) | 12.20 | - |
| [Dry](javascript:;) [matter](javascript:;) | 87.80 | - |
| [Crude](javascript:;) [protein](javascript:;) | 7.02 | 8.0 |
| [Acid detergent fiber](http://www.baidu.com/link?url=hNFDiwJvrBGZqABUSggfO6J3ZSbK_25UGHiCLQn6tkF5jD-oxnSJhQyXPcAAkQufmFbIWf5QSnB4ABbIDWoaMimOPNIpV3MzH-GNpViElxY1wIfQ_7dYfZMMBO5GwbBq) | 22.39 | 25.5 |
| Neutral detergent fiber | 27.52 | 31.34 |
| [Lignin](javascript:;) | 2.81 | 3.20 |
| [Non-fiber carbohydrate](http://www.baidu.com/link?url=CnweEG9Qd0OXO8EucEq997h3huIW-7zLRBsXrj4c8A-hX_NwSvkD3S2422VUqKaNMvh_GP4yHMtiLe3cnOrs8MiMO3h0b-mtFDAL32C3UK-1fOjrQg4SQzy5oxy0bjWg) | 41.31 | 47.05 |
| Starch | 22.13 | 25.2 |
| Fat | 0.09 | 0.10 |
| Ash | 11.86 | 13.51 |
| [Calcium](javascript:;) | 1.44 | 1.64 |
| [Phosphorus](javascript:;) | 0.43 | 0.49 |
| [Magnesium](javascript:;) | 0.32 | 0.37 |
| [Potassium](javascript:;) | 1.73 | 1.97 |
| [Sulfur](javascript:;) | 0.35 | 0.40 |
| [Chloride](javascript:;) | 1.19 | 1.36 |
