# Supplementary material for: Dietary Lasia spinosa Thw. improves reproductive performance of aged roosters
Source: Front Nutr. 2022 Aug 29;9:994783. doi: 10.3389/fnut.2022.994783 (PMC9466466; doi:10.3389/fnut.2022.994783)
Supplement: Supplementary Table 2 — Primers used for Real-time PCR. [file Table_2.DOCX]

**Table S2**

Primers used for Real-time PCR

| Gens | Forward primer (5′→3′) | Reverse primer (5′→3′) | Product size, bp | Accession number |
| --- | --- | --- | --- | --- |
| *β-actin* | CCAGCCATCTTTCTTGGGTA | ATGCCAGGGTACATTGTGGT | 141 | NM_205518.2 |
| *CASS4* | TCCCAAAAGGTTTGCCCACT | CTGTTTTTGCTGTCCGCACT | 172 | NM_001389548.2 |
| *ABCC2* | AATTACAGAGGGCACAGGGC | GGGTCCCAGGTGACGATGT | 81 | XM_025151804.3 |
| *LOC107057197* | GGAAGCCAGAAAGTCGTGGA | CTTCTGCTCCTGGACACTGG | 290 | XM_040650243.2 |
| *GSTA2* | GGCGCTGCAGTCAAGCTC | TCCTCGAATTCAACCCCAGC | 285 | NM_001001776.2 |
| *FOXB2* | ATGGCTCACTACTTCCACCC | ATGGCGAAGGGATGCTTGAA | 182 | XM_015280322.4 |
| *LOC107050176* | GCCGGGTTTACCCAATGAAG | CTGACCGGTGAGAATCGTGT | 95 | XM_046906082.1 |

*CASS4 =* Cas scaffold protein family member 4; *ABCC2 =* ATP binding cassette subfamily C member 2; *GSTA2 =* glutathione S-transferase alpha 2; *FOXB2 =* forkhead box B2.
